# Analysis of electrochemical impedance spectroscopy response for commercial lithium-ion batteries: modeling of equivalent circuit elements

**DOI:** 10.3906/kim-1910-72

**Published:** 2020-06-01

**Authors:** Uğur MORALI, Salim EROL

**Affiliations:** 1 Department of Chemical Engineering, Faculty of Engineering and Architecture, Eskisehir Osmangazi University, Eskisehir Turkey; 2 Department of Chemical Engineering, College of Engineering and Technology, American University of the Middle East, Egaila Kuwait

**Keywords:** Impedance spectroscopy, rechargeable battery, equivalent circuit, state of charge, Taguchi method

## Abstract

Electrochemical impedance spectroscopy measurements were performed to capture the physically meaningful parameters of commercially available 18650 cylindrical and 2032 coin cells by using the equivalent circuit model. The impedance response of the batteries was systematically investigated and discussed. A detailed analysis was achieved providing a determination of influential factors on the equivalent circuit parameters. The results suggested that the cell type tested here influenced the equivalent circuit elements profoundly. Taguchi analysis indicated that state-of-charge had the highest effect on the cathodic constant-phase-element exponent. The results contribute to full electrochemical analysis that is required for battery characterization.

## 1. Introduction

The growing population of the world and the reduction in conventional energy sources create an ever-increasing need for energy storage materials [1]. Rechargeable lithium-ion (Li-ion) batteries (LIBs) are currently admitted as the preferred technology for electrical energy storage and power delivery solutions [2]. LIBs are also a substantial technology for electric vehicles [3] and choices of this technology extend beyond portable electronics [4] to electric vehicles [5] and grid energy storage systems [6].

The power capability of a battery is intimately involved in its impedance characteristics [7]. The impedance of a cell is the potential drop under an alternative current and is related to the frequency and retention of the applied current [8]. There are multiple methods applied for impedance measurement, and there is no one-size-fits-all approach. These methods include pulse power test, electrochemical impedance spectroscopy (EIS), and pulsed multisine signal test [9]. EIS is a nondestructive method for Li-ion cells providing tracking for the evolution of LIBs [10]. Thus, this powerful technique enables diagnosis and prognosis for batteries.

Recent literature shows that there is a significant effort towards improvement of LIB properties to extend their use [6,7,9]. The battery characteristics associated with performance parameters depend on a wide range of factors such as temperature, cell type, cell constituents, applied current, state-of-charge, and cyclic aging [11]. Therefore, it is of great importance to elucidate the relationship between these factors and cell performance parameters.

The execution of EIS in improving models for the characterization of electrochemical properties for distinct battery systems has been seen in the literature. Wang et al. [12] used two different charge protocols at two different temperatures, 25 °C and 60 °C, to examine the influence of these factors on LIB degradation, while Schmidt et al. [2] employed high discharge rates to test power capability of cycled cells. In addition, Schmidt et al. conducted impedance tests to investigate the relationship between cell design and the aging route. In another study using a commercial Li-ion cell, Ovejas and Cuadras opted to measure the ohmic potential drop, activation polarization, and concentration polarization under low and medium discharge rates [13]. In a similar study by Matsuda et al., electrochemical impedance measurements were performed to examine the effects of temperature on the electrochemical features of commercial Li-ion cells after 700 cycles [3]. Erol and Orazem developed a novel process model to analyze the impedance response of Li-ion battery at different states-of-charge (SoC) and temperatures [11]. The developed process model fitted well to the impedance response, suggesting a detailed interpretation of the performance of commercial Li-ion cells. Moralı and Erol recently measured the impedances versus the state-of-charge of the commercial batteries to elucidate how the characteristics of the batteries under different SoCs are related to the alteration in cell parameters [7].

Numerous above-mentioned features of LIBs make performance parameter investigation more complicated. Furthermore, a wide variety of research efforts have compared the features of distinct LIBs and examined the effect of factors on performance parameters. In terms of optimization, the effective factors and their levels are often ill-determined in literature. More precise determination of influential factors can provide information to scientists interested in accurately identifying distinct factor effects on battery systems. Therefore, further research on battery optimization is essential. Understanding the precise effect of these factors on performance parameters may provide an avenue for Li-ion cells through effective optimization.

In this study, a comprehensive investigation of alternative current impedance changes as a function of state-of-charge and cell type was successfully made for a robust characterization of the commercially available high-power LIB cells by using the Taguchi design method. Since SoC and cell type are factors that can be controlled during the operation of batteries, comprehending how these two factors together affect cell properties is essential. That is why it would be interesting to investigate the parameters of LIBs at different SoC and cell geometry through Taguchi design.

## 2. Materials and methods

### 2.1. Experimental protocol

Commercial, rechargeable 18650 cylindrical (https://voltaplex.com ›sony-v3-18650-battery-us18650v3) and 2032 coin (www3.lenovo.com ›MSDS-QA-SDS601553) cell batteries used for EIS measurements were purchased from Sony Energy Devices Corporation. Their features are as follows: for 18650 cell, nominal voltage is 3.70 V, nominal capacity is 2600 mAh, maximum charge potential is 4.20 V, and discharge cut-off potential is 2.75 V. For 2032 cell, nominal voltage is 3.70 V, nominal capacity is 3500 mAh, maximum charge potential is 4.20 V, and discharge cut-off potential is 3.00 V. The 18650 notation represents that the cell was 18 mm in diameter and 65 mm in height. Similarly, the 2032 notation shows that the cell was 20 mm in diameter and 3.2 mm in height. The cathode was a lithium metal oxide, such as LiNiMnCoO2 , and the anode was graphitic carbon for both batteries. Battery holders (Gamry Instruments) for the cylindrical 18650 and coin 2032 cells were used to eliminate additional impedances due to cable connections.

Charge tests, as well as EIS measurements, were carried out with Gamry Reference 3000 Potentiostat/Galvonastat/ZRA connected to a desktop computer. Once the open circuit potential is adjusted, it should not change during EIS analysis. Therefore, the batteries were charged by applying standard constant current, and following the constant potential (CC-CP) procedure, as stated in the manufacturer’s specification, from 0% SoC to 100% SoC condition. In the CC-CP charge mode, the charge currents were 100 mA and 30 mA for the 18650 and 2032 cells, respectively. Dwell at the specified SoC levels was maintained while the current was allowed to decrease to 0.0001 A. All of the CC-CP steps had a 3-min rest between the charge step and EIS measurement. This procedure allowed the potential at the specific SoC to relax before EIS measurement. The battery was allowed to stabilize for a period of time for each potential step, in which the current was reduced to a value that was smaller than 0.1 mA. The amplitude should be adjusted based on a signal to noise ratio and conserve the linearity, playing an influential role in the accuracy of the result [14]. Thus, the cell impedance data were collected over a range of frequencies between 100 kHz and 0.01 Hz by applying a 10-mV alternating potential. Impedance scans were performed 3 times at the adjusted SoC. A Faraday cage was used to provide an effective protection from environmental electronic noise allowing for high quality electrochemical measurements, particularly at very low currents. The EIS data files were analyzed by running data analysis in Echem Analyst. The Simplex method was used to fit EIS data to the created equivalent circuit.

All electrochemical experiments were performed at room temperature and each experiment was repeated 3–4 times with the same type of battery to ensure that the results were both consistent and reproducible.

### 2.2. Equivalent circuit

**Figure 1:**
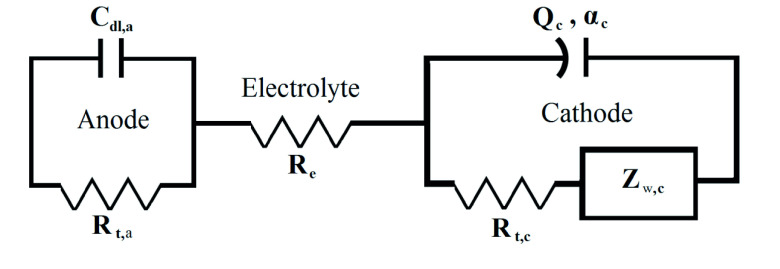
Equivalent circuit model created to extract the physically meaningful parameters of LIBs from EIS data.

An equivalent circuit was created by using the impedance model editor in Echem Analyst. A second-order equivalent circuit model was fitted to EIS data to extract the equivalent circuit model parameters of the cells. The created equivalent circuit is presented in Figure 1. The equivalent circuit model contains an electrolyte resistance Re , a charge transfer resistance for anode R_t,a_, a double layer capacitance for anode C_dl,a_, a constant phase element for cathode CPE_c_ (Q_c_, α_c_), a charge transfer resistance R_t,c_, and a Warburg impedance Z_w,c_. The intercalation/deintercalation of Li-ion is not uniform across the electrode surface due to the nonsmoothness of the electrode surface. This inhomogeneous structure of the electrode causes distinct charge transfer resistance and capacitive behavior because of the distribution of current on the electrodes. After all, the capacitive behavior of the cell is different from that of the double-layer capacitance. Therefore, a constant-phase-element (CPE) component was employed in the equivalent circuit for the cathode, as the CPE element describes the inhomogeneity of an electrode surface more accurately [15]. α_c_ in the equivalent circuit is the CPE_c_ exponent. The CPE_c_ exponent is highly related to a degree of surface inhomogeneity of the electrode and consequently to the distribution of current on the electrode surface. The diffusion of ions to the electrodes was represented by Z_w,c_. A full description of the mathematical model is provided in Moralı and Erol [7] and will therefore not be duplicated here.

### 2.3. Taguchi design of experiment

The Taguchi design was applied by using an orthogonal array to exhibit a robust optimization. The orthogonal array obtained the requisite data from the minimal number of experimental runs, and furthermore, it provided good separation of the optimum levels of each parameter for response variables. In this study, the input factors and their levels were selected based on our previous study [7]. The Taguchi design method was used to identify the factors having a significant influence on physically meaningful parameters. The orthogonal array was created using the Minitab17 statistical software. The matrix of experiments at various SoCs and cell types is represented in Table 1. The applied design method is outlined in our previous studies with more detailed information [16,17].

**Table 1:** Experimental design based on L16 orthogonal array.

Experiment	SoC	Type
1	0	cylindrical
2	0	coin
3	20	cylindrical
4	20	coin
5	30	cylindrical
6	30	coin
7	40	cylindrical
8	40	coin
9	60	cylindrical
10	60	coin
11	70	cylindrical
12	70	coin
13	80	cylindrical
14	80	coin
15	100	cylindrical
16	100	coin

## 3. Results

In this section, the impedance responses of 18650 and 2032 cells are presented for the selected SoC conditions and then results from the equivalent circuit model fitted to the EIS data are addressed. Finally, the results from the Taguchi design provide the optimum SoC and cell type for the equivalent circuit elements.

### 3.1. Analysis of impedance responses

**Figure 2:**
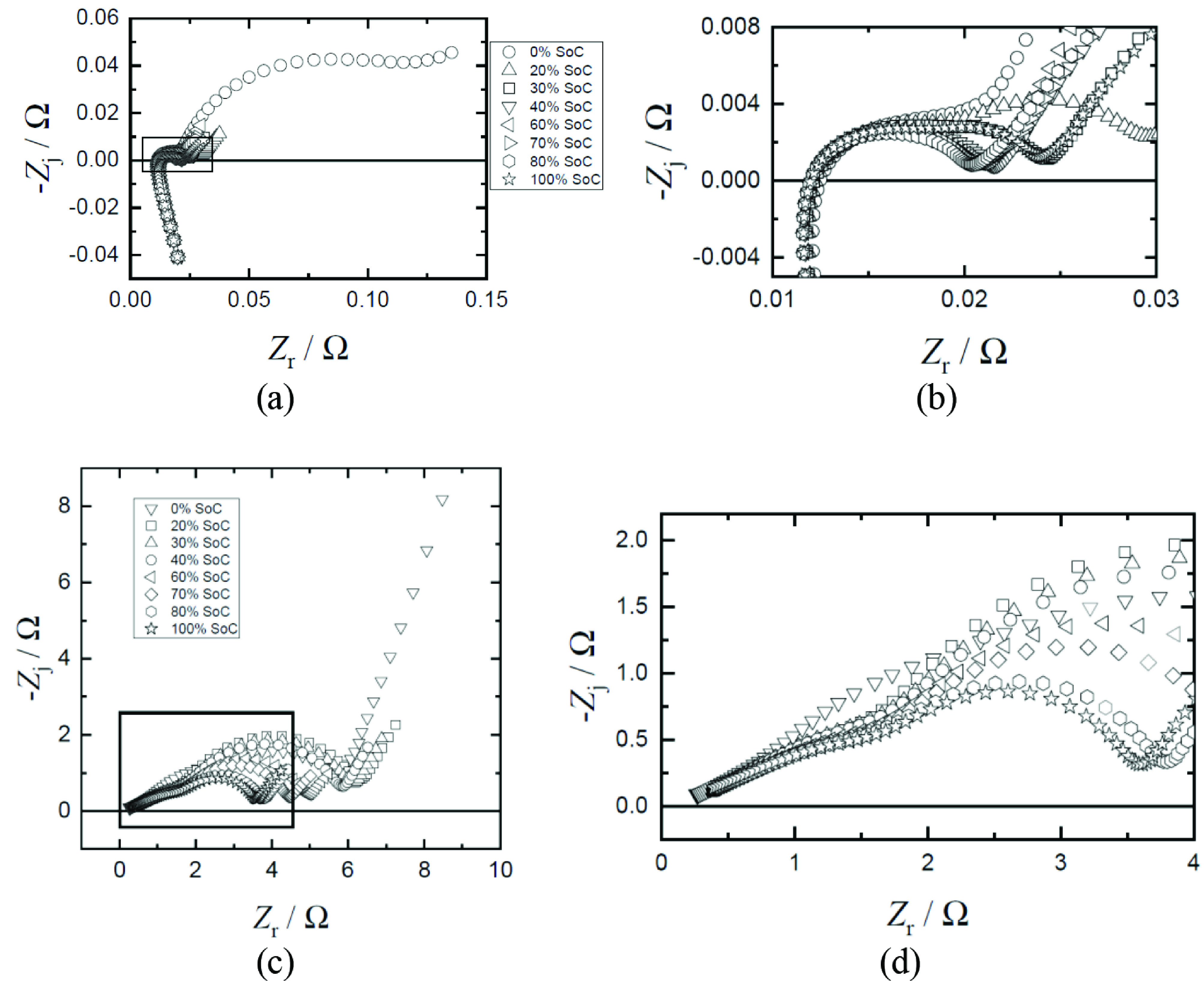
Equivalent circuit model created to extract the physically meaningful parameters of LIBs from EIS data.

The 18650 and 2032 cells were first charged following the CC-CP protocol to investigate how different SoC affects cell parameters. The relaxation step was applied to provide Li-ions a limited diffusion rate into the electrode in the CC-CP procedure. Thus, an applied current lower than the diffusion rate of Li-ions was expected to prevent the lithium plating on the electrode. After the CC-CP protocol, impedance measurements were performed under the desired cell potential conditions. The impedance response in Nyquist format of the 18650 cylindrical and 2032 button cells is shown in Figure 2.

The impedance behavior as a function of SoC is similar for the 18650 cylindrical and 2032 coin cells. The Nyquist plots in Figure 2 indicate that the second semicircle in the low-frequency region was more pronounced for the impedance data obtained at a low SoC (smaller than 20% SoC). For other potentials, the depressed semicircles were obtained at high and intermediate frequencies as SoC increased. The size of high-frequency semicircle stayed almost constant among all potentials. Only one semicircle in the middle-frequency region was observed in the spectra, especially around 40%–80% SoC. This semicircle could be associated with the electrochemical processes occurring at very close time constants on both the anode and the cathode. However, an elaborate analysis brought light to the presence of at least two semicircles overlapping. The parameters captured by fitting the model were discussed carefully because of the overlapping of the semicircles.

**Figure 3:**
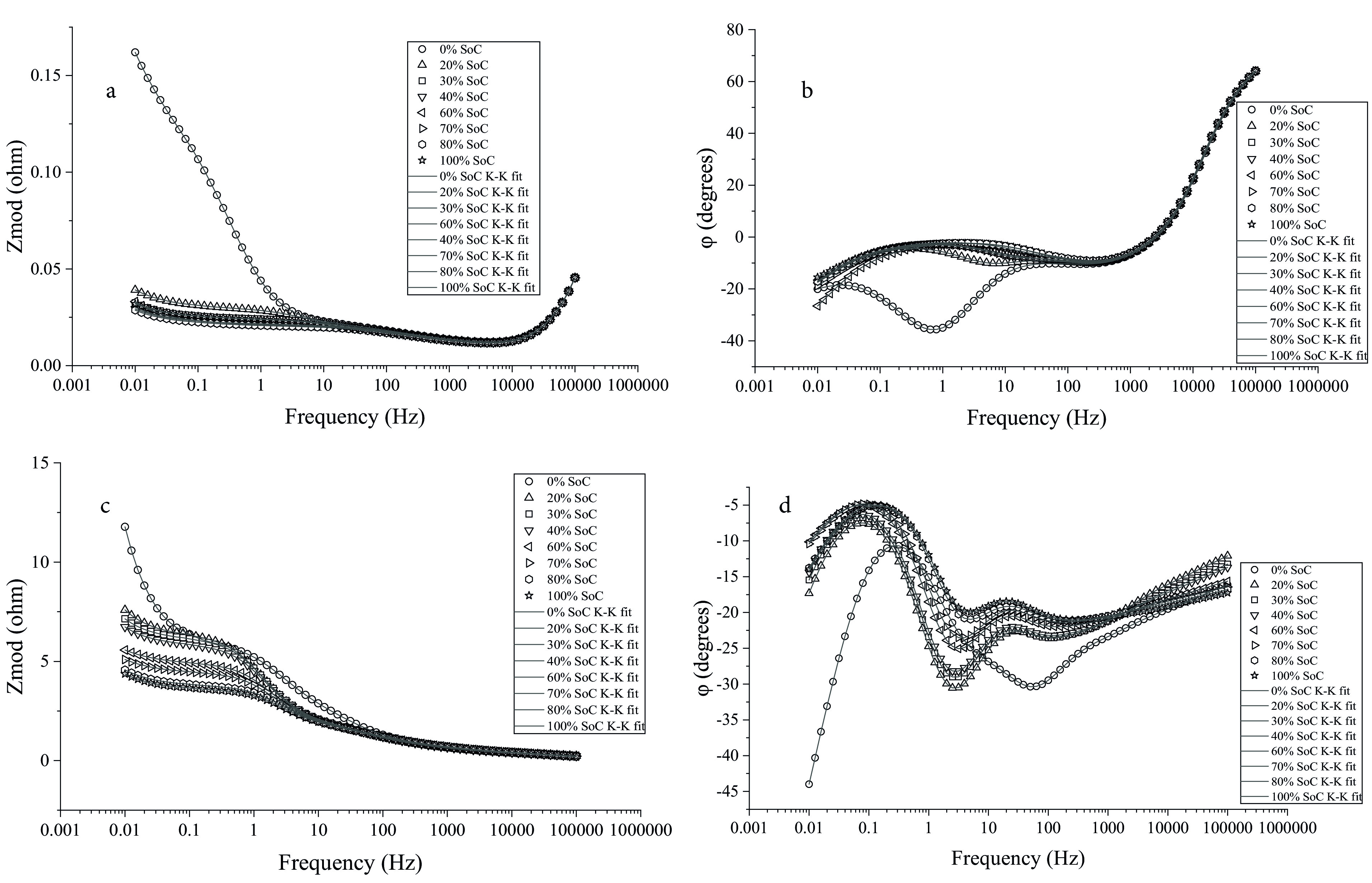
Impedance response in Bode format under normal potential range; a) |Z| vs. frequency for the 18650 cell; b) phase angle vs. frequency for the 18650 cell; c) |Z| vs. frequency for the 2032 cell; d) phase angle vs. frequency for the 2032 cell. These results provide a different representation of the data presented in Figure 2. The lines represent fits of Kramers-Kronig relations to the data.

The employed circuit elements provide information about ohmic resistance, transfer of Li-ions through solid-electrolyte interphase layers, and the resistance to charge-transfer occurring at electrode/electrolyte interface. The Simplex method was used to optimize the fit parameters. The parameter values of the elements in the model were adjusted to find the best fit. Physically meaningful parameters were extracted by using the Simplex method. The regressed model parameters are listed in Tables 2 and 3. The regression results for the data presented in Figure 2 are presented in Figure 3.

**Table 2:** Regression results of the 18650 cell and ±σ confidence intervals of fitting the equivalent circuit model to the EIS data over a potential range. The parameter calculation error was determined to be within 5% during the entire SoC of the batteries, meeting the parameter accuracy requirements set out in our study.

Parameter	0% SoC	20% SoC	30% SoC	40% SoC	60% SoC	70% SoC	80% SoC	100% SoC
*R_e_* / Ω	0.01304 ± 0.0001042	0.01276 ± 0.0001048	0.01268 ± 0.000105	0.01174 ± 0.0001424	0.01218 ± 0.0001158	0.01241 ± 0.0001056	0.01228 ± 0.0001025	0.01249 ± 0.0001033
*Q_c_* / F s^α-1^	6.645 ± 0.2328	3.523 ± 0.7063	1.807 ± 0.6748	1.506 ± 0.7467	0.3964 ± 0.09496	0.7297 ± 0.2238	0.8448 ± 0.2823	2.046 ± 0.6902
α	0.7941 ± 0.01596	0.8949 ± 0.06673	0.9987 ± 0.1161	0.8685 ± 0.1456	0.8342 ± 0.03718	0.8319 ± 0.1112	0.7909 ± 0.1002	0.9925 ± 0.1045
*W* / Ω s^1/2^	62.30 ± 4.577	282.5 ± 14.71	340.4 ± 16.38	361.9 ± 18.29	374.1 ± 30.03	329 ± 12.02	373.5 ± 14.96	359.3 ± 18.17
*R_t,c_* / Ω	0.1023 ± 0.004218	0.009499 ± 0.000732	0.004887 ± 0.0007936	0.004785 ± 0.0002542	0.007608 ± 0.0002642	0.007513 ± 0.001054	0.006782 ± 0.001353	0.005196 ± 0.0007125
*C_dl_* / F	0.1411 ± 0.009597	0.1374 ± 0.01071	0.1348 ± 0.01142	0.1157 ± 0.009753	0.1108 ± 0.006889	0.1128 ± 0.00951	0.1692 ± 0.01137	0.1408 ± 0.01183
*R_t,a_* / Ω	0.006569 ± 0.0002758	0.005740 ± 0.0004755	0.005484 ± 0.0006587	0.004163 ± 0.0002025	0.005021 ± 0.000330	0.004337 ± 0.0004874	0.00687 ± 0.0003542	0.005279 ± 0.0005744

**Table 3:** Regression results of the 2032 cell and ±σ confidence intervals of fitting the equivalent circuit model to the EIS data over a potential range. The parameter calculation error was determined to be within 5% during the entire SoC of the batteries meeting the parameter accuracy requirements set out in our study.

Parameter	0% SoC	20% SoC	30% SoC	40% SoC	60% SoC	70% SoC	80% SoC	100% SoC
*R_e_* / Ω	0.2979 ± 0.00374	0.4552 ± 0.004809	0.4636 ± 0.004989	0.4687 ± 0.005008	0.4799 ± 0.005272	0.4841 ± 0.005367	0.4807 ± 0.005377	0.474 ± 0.005347
*Q_c_* / F s^α-1^	0.01357 ± 0.001109	0.03117 ± 0.001496	0.03224 ± 0.001586	0.03266 ± 0.001655	0.03516 ± 0.002005	0.03503 ± 0.002193	0.03105 ± 0.002451	0.03279 ± 0.00273
α	0.7223 ± 0.02199	0.8839 ± 0.02196	0.8583 ± 0.02153	0.8771 ± 0.02365	0.8645 ± 0.02735	0.8756 ± 0.03006	0.9048 ± 0.03821	0.9062 ± 0.04049
*W * / Ω s^1/2^	0.5262 ± 0.01118	1.695 ± 0.1032	2.073 ± 0.1478	2.316 ± 0.1737	3.634 ± 0.3552	3.759 ± 0.3409	3.121 ± 0.1996	3.212 ± 0.2016
*R_t,c_* / Ω	4.184 ± 0.04602	4.545 ± 0.05283	4.464 ± 0.05156	4.152 ± 0.04861	3.406 ± 0.04080	2.946 ± 0.0366	2.240 ± 0.03057	2.064 ± 0.02919
*C_dl_* / F	0.0001588 ± 0.000005986	0.0007863 ± 0.00002046	0.0007193 ± 0.00001896	0.0007245 ± 0.00004861	0.0006389 ± 0.0000172	0.0006107 ± 0.00001668	0.0005909 ± 0.00001616	0.0005715 ± 0.00001548
*R_t,a_* / Ω	0.3746 ± 0.007614	0.8712 ± 0.0128	0.8777 ± 0.01301	0.9075 ± 0.01310	0.8746 ± 0.01269	0.8617 ± 0.01246	0.8509 ± 0.01224	0.8532 ± 0.01206

Ohmic resistance is an important parameter for batteries. The increase in ohmic resistance is one of the causes of temperature rise and thermal runaway that might occur in the cells [18]. The intersection of EIS data with the axis indicating the real part of impedance, which corresponds to ohmic resistance, increased as SoC of the 2032 cell increased from 0% to 20%. After 20% SoC, the slight increase in SoC of the 2032 cell slightly increased the electrolyte resistance. A slight decrease in R_e_ was observed when SoC was 100%. The trend for R_e_ vs. SoC was different for the 18650 cells. The electrolyte resistance of 18650 cell decreased as SoC increased from 0% to 40%. After 60% SoC, the R_e_ of 18650 cells did not change excessively in the next potentials. The differences in ohmic resistances of the cells indicated separate features of the electrolyte solutions in these cells. Compared with 2032, 18650 had a lower ohmic resistance, suggesting better performance of the 18650 cells. The distinct electrolyte resistances indicated a difference in the relationship between electrolyte and Li-ions in the 18650 cylindrical cells and those in 2032 coin cells.

The resistance in the electron transfer process occurring across different layers is the charge-transfer resistance [11]. The charge transfer resistance is generally associated with the reaction at the electrode/electrolyte interface where the charge is transferred. According to the above-mentioned information, the charge transfer resistance could change with the variations in the electrochemical reactions occurring on the electrodes. The cathodic charge transfer resistance, R_t,c_, of the 2032 cell was higher at lower SoC, indicating large potential polarization at low potentials [19]. The decrease in R_t,c_ of the 2032 cell from 30% SoC to 100% SoC could be attributed to the higher number of available Li-ions in the cathode region. In the case of 18650 cell, the cathodic resistance was at the highest value at 0% SoC. However, there was no tendency of increasing or decreasing for R_t,c_ of the 18650 cell. A 0% SoC showed a drastic decrease for the R_t,c_ as the SoC increased to 20% SoC and then to 40% SoC. Between 60%–70% SoC, the alteration in the cathodic charge-transfer resistance was not observed significantly. Even though R_t,c_ values constantly stayed at low SoCs, they changed dramatically at higher than 50% SoC. In contrast to the cathodic charge-transfer resistances, the anodic charge-transfer resistances (R_t,a_) did not depend strongly on cell potential or SoC. Compared with 2032 cell, the 18650 cell exhibited a lower charge transfer resistance than the 2032 cell. This result was indicative of the higher performance characteristic of 18650 batteries.

Compared to the anodic capacitance C_dl,a_, the cathode side exhibited a higher capacitance value for the 2032 cell. This behavior was also similar to the 18650 battery. However, both anodic and cathodic capacities of 2032 cells were lower than 18650 cells. The results showed that the capacitance parameter is independent of SoC. The cathodic capacitance (Q_c_) of 2032 cells decreased with an increase in SoC (up to 50% SoC), while the Q_c_ of 18650 cells showed this trend up to 60% SoC. Similar to cathodic capacitance, the anodic capacitance of 2032 cells decreased (from 20% SoC up to 100% SoC) when SoC increased. For 18650 cells, the relationship between C_dl,a_ and SoC was similar to the 2032 cell up to 70% SoC. Electrochemically active materials in the cells could influence the capacitance of the electrodes [20]. The change in electrode capacitances could be attributed to the oxidation-reduction reactions occurring in the cells. Under different SoC conditions, the side reactions taking place in the electrolyte/electrode interface may have caused the changes in electrode capacitances [19]. According to the above-mentioned possibilities, the decrease in the amount of active material available for intercalation and the inactive species precipitation consuming Li-ions may have led to changes in electrode capacitances.

CPE exponent is only deemed reasonable if its value is between 0.5–1.0. The cathodic CPE exponent (α_c_) enables estimation of the roughness of the electrode surface. The cells under all SoC conditions showed high values of αc , close to 1.0. The higher cathodic CPE exponent indicated the smooth surface of the electrodes, and therefore, the uniform distribution of current on the cathode. Compared with the 18650 cells, the 2032 cell showed lower α_c_ values, indicating a surface texture of less smooth surface. For both batteries, the results showed that α_c_ tended toward unity at larger SoC. The relationship between α_c_ and SoC showed that the diffusion of Li-ion was more difficult at lower SoC. Similar conclusions were recently made by Moralı and Erol [7], who showed that cathodic CPE exponent increased at higher SoC.

### 3.2. Taguchi design

The approach in this study allows for the identification of optimum conditions for SoC and cell type without an increased number of experiments and time. The equivalent circuit described the impedance response of the batteries. Each element of the circuit had physically meaningful parameters for the 18650 and 2032 cells. In the Taguchi method, equivalent circuit elements were investigated as the response variable. If the response variable is desired to be the smallest value, the smaller-is-better signal-to-noise (S/N) ratio was used. For quality assurance of the response variable, the larger-is-better S/N ratio was applied to maximize the response variable. The smaller-is-better S/N ratio was applied to R_e_, R_t,a_, and R_t,c_, representing electrolyte resistance, charge transfer resistance for the anode, and charge transfer resistance for the cathode, respectively. The larger-isbetter S/N ratio was applied to C_dl,a_, Q_c_, and α_c_, representing double-layer capacitance for anode, capacitance for cathode, and cathodic CPE exponent, respectively. The applied orthogonal array of L16 enabled analysis of the entire parameter space. Thus, the optimum level of each factor was attained for the response variable to be maximized or minimized. The S/N ratios of each level of both SoC and cell type are presented in Table 4. This type of table is identified as the response table in the Taguchi design [17]. The response table presented in Table 4 consists of S/N ratio, delta, and rank. Delta was calculated by subtracting the smallest S/N ratio from the largest S/N ratio. As an example, for the electrolyte resistance R_e_, the highest S/N ratio of SoC was 24.107 and the lowest one was 22.213. Thus, the delta of SoC was calculated to be 1.894. Rank was used to provide convenience to determine which factor has the largest influence on the response characteristic. Rank 1 indicated the importance of the factor. The factor with rank 1 was more effective on the response variable than the factor with rank 2. The optimum level of the factors was determined based on the S/N ratios. The level with the highest S/N ratio indicated the optimum condition for the factor. Level 1 of both SoC and cell type was the optimum conditions for lower electrolyte resistance. Similarly, the best conditions for Ra were the first level of both SoC and cell type. Level 8 of SoC and level 1 of cell type were the best conditions to attain the lowest R_t,c_. In the case of the highest C_dl,a_ , level 2 and level 1 were the optimum conditions for SoC and cell type, respectively. These levels of SoC and cell type also optimized Q_c_. For the cathodic CPE exponent α_c_, level 8 of SoC and level 1 of cell type maximized the α_c_ parameter. Additionally, for all other response variables except α_c_, the battery type was a more influential factor than SoC. In other words, the variation of α_c_ with SoC was more than in the case of cell type. The factor SoC only had more efficient on the α_c_ than the cell type. Thus, the influence of both SoC and cell type on the equivalent circuit elements was analyzed based on Table 4 in greater detail.

**Table 4:** 

	S/N ratio for R_e_		S/N ratio for R_a_		S/N ratio for R_c_		S/N ratio for C_dl_		S/N ratio for Q_c_		S/N ratio for α_c_	
Level	SoC	Type	SoC	Type	SoC	Type	SoC	Type	SoC	Type	SoC	Type
1	**24.107**	**38.102**	**26.089**	**45.419**	3.685	**40.849**	--46.5	-- **17.61**	--10.449	**3.758**	--2.4141	-- **1.1849**
2	22.359	7.017	23.01	2.118	13.648	--10.534	-- **39.66**	--65.17	-- **9.594**	--30.632	--1.0182	--1.3134
3	22.307		23.176		16.612		--40.13		--12.346		--0.6693	
4	22.594		24.227		17.019		--40.77		--13.082		--1.1818	
5	22.332		23.574		15.865		--41.5		--18.558		--1.4196	
6	22.213		24.275		16.55		--41.62		--15.924		--1.3762	
7	22.289		22.332		18.184		--40		--15.812		--1.4533	
8	22.277		23.464		**19.696**		--40.94		--11.734		-- **0.4605**	
Delta	1.894	31.085	3.758	43.301	16.011	51.382	6.83	47.55	8.965	34.39	1.9536	0.1285
Rank	2	1	2	1	2	1	2	1	2	1	1	2

The optimum levels are highlighted in bold.

The analysis of variance (ANOVA) results based on a significance level of 0.05 are presented in Table 5. The P-value provided a statistical determination of the importance of the factors. A P-value smaller than 0.05 indicated significant factors for the desired value of the response variable. The results indicated that there was a statistically significant association between the cell type and the entire response variable, except for α_c_. A P-value larger than 0.05 suggested that the effect of SoC was not statistically significant. The results showed that equivalent circuit elements were not affected by all SoC conditions. The results also indicated that statistically there was no relationship between the CPE exponent, and cell type was visible. A lower P-value for SoC than for cell type denoted that SoC was more influential on α_c_. Statistically, SoC had almost no influence on the other response variables.

**Table 5:** ANOVA table for equivalent circuit elements.

R_e_	DF	Seq SS	Adj SS	Adj MS	F	P		C_dl_	DF	Seq SS	Adj SS	Adj MS	F	P
SoC	7	0.013523	0.013523	0.001932	0.98	0.508		SoC	7	0.001324	0.001324	0.000189	0.99	0.505
Type	1	0.767604	0.767604	0.767604	390.86	0.000		Type	1	0.069934	0.069934	0.069934	366.37	0.000
Error	7	0.013747	0.013747	0.001964				Error	7	0.001336	0.001336	0.000191		
Total	15	0.794874						Total	15	0.072593				
														
R_a_	DF	Seq SS	Adj SS	Adj MS	F	P		Q_c_	DF	Seq SS	Adj SS	Adj MS	F	P
SoC	7	0.10827	0.10827	0.01547	0.99	0.506		SoC	7	14.617	14.617	2.088	0.99	0.506
Type	1	2.5824	2.5824	2.5824	165.05	0.000		Type	1	18.607	18.607	18.607	8.8	0.021
Error	7	0.10952	0.10952	0.01565				Error	7	14.802	14.802	2.115		
Total	15	2.80019						Total	15	48.025				
														
R_c_	DF	Seq SS	Adj SS	Adj MS	F	P		α_c_	DF	Seq SS	Adj SS	Adj MS	F	P
SoC	7	3.5107	3.5107	0.5015	1.04	0.480		SoC	7	0.047537	0.047537	0.006791	2.04	0.185
Type	1	48.4849	48.4849	48.4849	100.5	0.000		Type	1	0.000798	0.000798	0.000798	0.24	0.640
Error	7	3.3771	3.3771	0.4824				Error	7	0.023357	0.023357	0.003337		
Total	15	55.3726						Total	15	0.071693			

## 4. Discussion

In this study, electrochemical impedance spectroscopy combined with Taguchi design offered an unprecedented insight into the equivalent circuit parameters of the commercially available Li-ion cells, allowing a much better understanding of the influential levels of these factors. The Taguchi technique combined with EIS is believed to be valuable to the reader and to researchers in developing experimental procedures for Li-ion batteries. The results suggested that the cell type tested here had a profound impact on the equivalent circuit elements, whereas SoC only had a higher effect on the cathodic CPE exponent. It can be concluded that if the goal is to decrease the charge-transfer resistances without a decrease in capacitances, commercially available 18650 cells could be preferred for an efficient energy system. The Taguchi design method opened the door for the full analysis requiring battery characterization. Therefore, an orthogonal array of different factors in further studies would be beneficial for the modeling of lithium-ion batteries under different conditions in real applications.
